# The Consequence of the Cape: A Mixed-Methods Inquiry into the Sociocultural Stressors Impacting Black Women’s Health

**DOI:** 10.1007/s40615-025-02636-x

**Published:** 2025-09-22

**Authors:** Samantha R. Jones, Debbynie Barsh, Lillie D. Williamson, Kathleen McIntyre, Christy J. W. Ledford

**Affiliations:** 1 Department of Family and Community Medicine, Medical College of Georgia, Augusta University, 1120 15th St, Augusta, GA 30912 USA; 2 Department of Communication Arts, University of Wisconsin-Madison, 500 Lincoln Dr, Madison, WI 53706 USA

**Keywords:** Health disparities, Racial disparities, Chronic stress, Sociocultural stress, Intersectionality, Healthcare inequities

## Abstract

**Background:**

Black women face a disproportionate burden of health issues, including higher rates of aggressive breast cancer and chronic stress–related conditions. This study examines how sociocultural identity and stressors impact health outcomes among Black women.

**Methods:**

Using a convergent mixed-methods design, we analyzed qualitative data from in-depth interviews with 50 Black women aged 18–49 and quantitative data from a similar subset of respondents to the 2022 Health Information National Trends Survey (HINTS). Integrated analysis mixed Sort and Shift, Think and Shift qualitative analysis with analyses of covariance from the quantitative data.

**Results:**

Through integrated analysis, we developed a model of how sociocultural identities and experiences of Black women impact their health. The resulting quantitative analysis showed that race was associated with an increased risk of depressive symptoms. Women who reported worse feelings about income, greater social isolation, or less meaning and purpose also scored higher with depressive indicators.

**Conclusion:**

The intersectional stressors Black women face contribute to adverse health outcomes, underscoring the need for culturally sensitive health interventions and policies that address the specific identity-based stressors affecting this population. Recognizing and addressing these unique stressors are essential steps for healthcare providers and policymakers to improve health equity. Such efforts are vital in reducing health disparities, especially in relation to aggressive breast cancer and other chronic conditions that disproportionately impact Black women.

## Introduction

Black women account for 13.6% of the US female population but face disproportionate health burdens, including higher prevalence and mortality rates for several chronic conditions [1, 2]. Among these, breast cancer—particularly aggressive subtypes like triple-negative breast cancer—disproportionately affects Black women under the age of 50, who experience a mortality rate twice that of White women in the same age group [3–7]. Despite improvements in screening and treatment over the last four decades, disparities in survival persist—pointing to the need for deeper investigation into the underlying contributors to risk and outcomes [8].

One area gaining increased attention is the role of chronic stress in shaping health trajectories [9–13]. Research suggests that sustained exposure to stress may contribute to inflammation, immune dysfunction, and other biological processes linked to cancer progression and recurrence [14–19]. However, limited research has examined how Black women understand and navigate the stressors that may affect their health, particularly during early to mid-adulthood when health risks begin to accumulate [20–22].

Existing racial health disparities may be partly attributed to long-standing structural inequalities, particularly in regions with high levels of structural racism [23–25]. Structural racism refers to the ways societies systemically reinforce racial inequity through institutions like housing, education, employment, healthcare, and media [26]. Examples of structural inequities include greater exposure to environmental pollutants, reduced access to fresh food, lower-quality healthcare systems, and limited safe recreational space—all of which contribute to chronic stress and shape health behavior patterns [27–30].

In addition to environmental and healthcare-based disparities, Black women face documented inequities across the workplace, family, and community domains. In the labor market, they earn substantially less than White men and are overrepresented in underpaid and inflexible occupations that offer limited benefits and protections [31–33]. At the same time, Black women are more likely to be primary or co-breadwinners while also providing extensive caregiving for children and elders within extended family networks [34, 35]. Community-level disparities such as neighborhood disinvestment and reduced access to essential services further compound these pressures [36]. These systemic conditions do not assume a uniform experience of stress, but they do help contextualize why these life domains may represent important environments for stress exposure over time. Scholars have theorized that individuals who navigate these intersecting structural forces may encounter complex and compounding forms of stress [37–40], yet more research is needed to understand how Black women experience and interpret these stressors across various aspects of daily life.

Examining the distinct stressors faced by Black women can provide a deeper understanding of how these experiences differ from those of other groups (e.g., White women or Hispanic women) and shed light on how sociocultural factors may play a role in the link(s) between stress, coping mechanisms, and health outcomes. By capturing these unique experiences, this research will contribute valuable insights into the factors that perpetuate health disparities among Black women. Few studies to date have integrated population-level data with in-depth qualitative narratives to examine how sociocultural identity influences stress and health outcomes among Black women, particularly in the context of structural racism and gendered expectations. This study applied mixed-methods to richly explain (1) the sociocultural identity and stressors of Black women in the community, workplace, and family and (2) how identity and stressors are connected to health outcomes.

## Methods

**Fig. 1:**
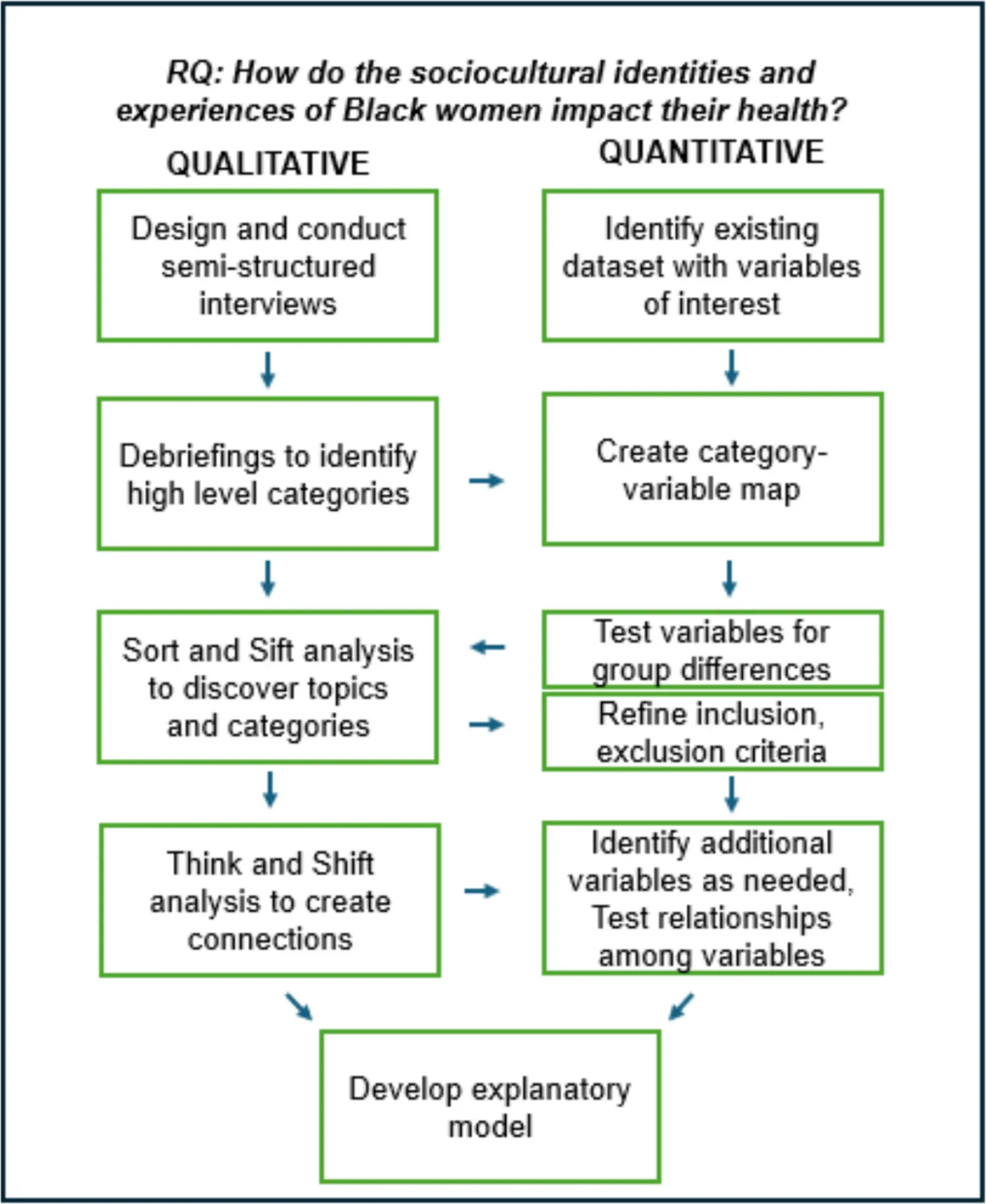
Mixed-methods convergent design flow chart

We conducted a convergent mixed-methods design to leverage the strengths of qualitative in-depth data in conjunction with secondary analysis of a large quantitative survey to gain a more comprehensive understanding of the sociocultural identity and stressors of Black women and how those identities and stressors are connected to health outcomes. See Fig. 1 for a flow chart illustrating the mixed-methods design. We applied a grounded theory approach to develop a model of the effects of sociocultural identity and stressors on health grounded in data from the rich descriptions of Black women’s perspectives and experiences. A team of female researchers, including those extensively trained in qualitative and mixed-methods, designed and implemented the study.

### Qualitative Phase

The qualitative phase included semi-structured interviews. AUTHOR1 and AUTHOR5 designed the interview guide to capture rich descriptions of women’s perspectives and experiences. See Box 1 for an overview of the interview guide. AUTHOR5 trained five women interviewers (research faculty, full-time research staff, medical students); three identify as Black or African American, one as White American, and one as Asian American. The Augusta University Institutional Review Board approved the qualitative phase.

**Table:** 

Box 1 Semi-structured interview guide	
Overarching lines of inquiry • How do Black women describe stress, specifically exploring what stress experiences they perceive as unique to Black female identity? • How do Black women describe the social and cultural expectations of womanhood? • How do Black women describe that socio-cultural expectations of womanhood affect their biopsychosocial health? • What coping behaviors do Black women describe they enact in response to socio-cultural expectations and pressure?	
Interview questions with sample follow-up questions from the interviews What comes to mind when you think about breast cancer, or cancer in general? Sample follow-up: Have you known anyone who has suffered from breast cancer? Tell me about how you think about your health Sample follow-up: What decisions do you make each day about your health? Describe your neighborhood to me Sample follow-up: How do you feel about the safety in your neighborhood? Tell me about your family Sample follow-up: How do you share household responsibilities? Tell me about your (grand)children’s schools Sample follow-up: Who do you see influencing your children? What does your average day look like? Sample follow-up: What is your job title? How do you feel about your finances? Sample follow-up: How is money a source of stress? Tell me about your social life Sample follow-up: How often do you spend time with family or friends? What are the pressures of living as a Black woman? Sample follow-up: Do you think this experience is unique to Black women? Tell me about how your identity as a Black woman has affected how you are treated by co-workers or customers Sample follow-up: How did you cope with that experience? Is there anything else that we haven’t discussed that you feel is important to the topic of Black women and stress, and health?	

#### Procedures

We recruited Black women, ages 18–49 (focusing on younger Black women to understand the experiences of this group who suffer from racial disparities in prevalence as presented in the introduction), through tabling at church and community events and posting flyers at factory workplaces, health clinics, and retail stores, along the border of Georgia and South Carolina. Interviews occurred in person (in private spaces accessible to community members) or virtually (via a university-licensed videoconferencing platform) at times convenient to the interviewee (Mondays through Saturdays, from 9 a.m. to 8 p.m.) from May 2023 to August 2024.

As grounded theory, we pursued theoretical saturation [41]. We reached this point when the categories were fully developed and model linkages were well described throughout the participants’ stories. In line with grounded theory, we did not set an a priori theoretical sample size [41–45], but we did recognize that literature suggests that a sample size of 30 or more participants is generally sufficient to claim theoretical saturation [46, 47].

During the consent process, interviewers described the study purpose and confidentiality protections. Interviewers asked women to create pseudonyms for data management, data analysis discussions, and reporting. This process empowers interviewees and maintains their personhood and avowed identity while maintaining anonymity [48]. After women provided verbal consent and permission to record, interviewers began the interview.

After each interview, interviewers wrote episode profiles, outlining initial reactions to develop a holistic view of the interview. Researchers met for a structured debrief, recorded through field memos, about interview logistics, how the interview guide worked and any needed revisions, emerging topics of interest, and future recruitment. All interview recordings were transcribed, and interviewers reviewed transcripts for accuracy and removed identifiers.

#### Qualitative Analysis

Throughout data collection and analysis, AUTHOR1, AUTHOR2, AUTHOR4, and AUTHOR5 (two Black women and two White women) met weekly to conduct analysis, using the Sort and Sift, Think and Shift method [49]. The team began with case-based analysis and developed a list of primary topics, which allowed researchers to look deeply into each case, gaining a sense of content, dimensions, and properties. After analyzing 11 transcripts together, AUTHOR4 analyzed all data (i.e., transcripts, field memos, and episode profiles) iteratively. The codebook was established using an iterative process, connecting themes, and establishing main concepts. Stepping back, AUTHOR4 diagrammed each case to elucidate important categories and identify recurring topics. Throughout this process, she presented case diagrams to the team to discuss the primary topics, dimensions, and properties, and the team monitored for larger themes that threaded throughout the data. Multiple verification strategies ensured trustworthiness of the data, including maintaining memos, reflexivity/responsivity, maintaining methodological coherence/congruence, and rich description of findings [50].

### Quantitative Phase

The quantitative phase included data from the 2022 Health Information National Trends Survey (HINTS6). HINTS provides nationally representative data, which has been collected by telephone biennially since 2003 [51]. HINTS6 was collected from March to November 2022 and contained 6252 responses for a response rate of 28.07%. Westat Institutional Review Board (IRB) designated HINTS 6 as “exempt research” under 45 Code of Federal Regulations (CFR) 46.104.

#### Quantitative Analysis

To align the HINTS6 quantitative data with demographics of the qualitative sample, we included only cases who reported they were “born female,” within the ages 18–49, and located in Census Region South in the data analysis. We then limited inclusion to respondents from racial and ethnic groups with at least 100 respondents: Hispanic American, non-Hispanic Black American, and non-Hispanic White American.

We planned analyses of variance to test for group differences. All statistical analysis was performed using Statistical Package for the Social Sciences (SPSS) 28 [52].

### Integrated Analysis

Figure 1 illustrates the integrated analysis flow. After 37 completed interviews, we reviewed all field and interview debriefing notes to identify high-level categories in the qualitative data. We compared this category list to the HINTS6 variable list to create a category-variable map. As qualitative analysis continued, we tested quantitative variables for group differences. From the qualitative Sort and Sift analysis, we refined inclusion and exclusion criteria to apply to the quantitative dataset. These preliminary group differences were incorporated into the interpretation of qualitative data. After completing 50 interviews, we advanced to Think and Shift analysis to create connections among the categories. This analysis informed which variables to include in the final quantitative analysis. See Table 1 for the concepts and operationalization. We also considered the qualitative findings when excluding cases for quantitative analysis. As a result, we case-wise deleted respondent cases with missing marital status and excluded 12 widows. We developed the emerging model through the integration and interpretation of both qualitative and quantitative data. Model refinement continued through the writing and revision of this manuscript.

**Table 1:** HINTS6 variables and group differences mapped within qualitative categories

Qualitative category						
Quantitative: HINTS6 Variables and operationalization	Scale reliability	Total (***n*** = 452)	NH Black (***n*** = 133)	NH White (***n*** = 199)	Hispanic (***n*** = 120)	Sig
Enculturated weights and stereotypes						
Ethnic belonging: Single item, measured on 5-level Likert agreement (1–5, where 1 is the strongest) *I have a strong sense of belonging to my own ethnic group*	-	2.14 (sd 1.12)	1.59 (sd 0.86)	2.48 (sd 1.13)	2.19 (sd 1.11)	*p* <.001
Life stressors						
Patient-centered communication (PCC) (opposite of illness invalidation): The PCC Scale transforms the mean value linearly of 7 items to a 0–100 scale (where 100 is best) *In the past 12 months, how often did your doctors, nurses, or other health professionals…* *… give you the chance to ask all the health-related questions you had?* *… give the attention you needed to your feelings and emotions?* *… involve you in decisions about your healthcare as much as you wanted?* *… make sure you understood the things you needed to do to take care of your health?* *… explain things in a way you could understand?* *… spend enough time with you?* *… help you deal with feelings of uncertainty about your health or healthcare?*	α =.964	66.80 (sd 33.03)	64.92 (sd 34.69)	71.55 (sd 28.52)	60.95 (sd 37.06)	n.s
Income feelings: Single item, 4 levels *Which one of these comes closest to your own feelings about your household income?* (1 = living comfortably on present income, 2 = getting by on present income, 3 = finding it difficult on present income, 4 = finding it very difficult on present income)	-	2.03 (sd 1.00)	2.20 (sd 1.04)	1.91 (sd 0.97)	2.04 (sd 1.04)	*p* <.05
Protective factors						
Meaning and purpose: *T*-score created from 4 items with 5 response options (very much, quite a bit, somewhat, a little bit, not at all). A higher score indicates greater meaning and purpose *My life has meaning* *I have a clear sense of direction in life* *I experience deep fulfillment in my life* *My life has purpose*	α =.874	54.71 (sd 10.27)	56.94 (sd 9.57)	53.39 (sd 9.83)	54.49 (sd 11.32)	*p* <.01
Social isolation (opposite of social support): *T*-score created from 4 items with 5 response options (never, rarely, sometimes, usually, always). A higher score indicates greater social isolation *I feel left out…* *I feel that people barely know me…* *I feel isolated from others…* *I feel that people are around me but not with me…*	α =.918	48.57 (sd 10.30)	48.93 (sd 11.34)	48.40 (sd 9.15)	48.47 (sd 10.94)	n.s
Health impact						
General health: Single item, with 5 response options. A higher score indicates worse health *In general, would you say your health is…* (excellent, very good, good, fair, poor)	-	2.61 (sd.98)	2.64 (sd.94)	2.47 (sd 1.03)	2.80 (sd.90)	*p* <.05
Patient Health Questionnaire (PHQ) 2 (depression): Total score of 2 items with 4 response options (nearly every day, more than half the days, several days, not at all). A higher score indicates greater depressive symptoms *Over the past 2 weeks, how often have you been bothered by…* *… little interest or pleasure in doing things?* *… feeling down, depressed, or hopeless?*	α =.749	1.45 (sd 1.63)	1.63 (sd 1.64)	1.25 (sd 1.56)	1.59 (sd 1.71)	n.s

## Results

We completed interviews with 50 Black women who met the inclusion criteria. See Table 2 for individual characteristics. Among the 50 interviews, 38 were completed in person and 12 on the videoconferencing platform. The average interview lasted approximately 68 min in length, for a total of 3418 min of interview data and 1375 transcribed pages. Table 2 summarizes individual characteristics for the 452 cases used in the analysis.

**Table 2:** Qualitative and quantitative sample characteristics

Characteristic	Interviewees (*n* = 50)	HINTS 6 respondents			
		**Total (*n* = 452)**	**NH White (*n* = 199)**	**NH Black (*n* = 133)**	**Hispanic (*n* = 120)**
Age					
18–26	8 (16%)	78 (17.3%)	36 (18.1%)	14 (10.5%)	28 (23.3%)
27–39	24 (48%)	212 (46.9%)	88 (44.2%)	68 (51.1%)	56 (46.7%)
40–49	18 (36%)	162 (35.8%)	75 (37.7%)	51 (38.3%)	36 (30%)
Employment					
Employed/student	46 (92%)	360 (79.6%)	159 (79.9%)	111 (83.5%)	90 (75%)
Retired	2 (4%)	2 (0.4%)	1 (0.5%)	1 (0.8%)	0 (0%)
Unemployed	2 (4%)	89 (19.7%)	38 (19.1%)	21 (15.8%)	30 (25%)
*Missing*	–	1 (0.2%)	1 (0.5%)	–	–
Marital status					
Married/living w. partner	15 (30%)	243 (53.8%)	132 (66.3%)	44 (33.1%)	67 (55.8%)
Single/never married	31 (62%)	156 (34.5%)	46 (23.1%)	76 (57.1%)	34 (28.3%)
Divorced/separated	4 (8%)	53 (11.7%)	21 (10.6%)	13 (9.8%)	19 (15.8%)
Total people in the household					
1	9 (18%)	64 (14.2%)	28 (14.1%)	27 (20.3%)	9 (7.5%)
2–3	30 (60%)	234 (51.8%)	106 (53.3%)	68 (51.1%)	60 (50%)
4 or more	11 (22%)	152 (33.6%)	64 (32.2%)	38 (28.3%)	50 (41.7%)
*Missing*	–	2 (0.4%)	1 (0.5%)	–	1 (0.8%)
Children in HH < 18					
0	23 (46%)	199 (44%)	103 (51.8%)	55 (41.4%)	41 (34.2%)
1–2	24 (48%)	198 (43.8%)	72 (36.2%)	63 (47.4%)	63 (52.5%)
3 or more	3 (6%)	50 (11.1%)	23 (11.6%)	14 (10.5%)	13 (10.8%)
*Missing*	–	5 (1.1%)	1 (0.5%)	1 (0.8%)	3 (2.5%)
Education^a^					
HS or less		93 (20.6%)	34 (17.1%)	26 (19.5%)	33 (27.5%)
Vocational/technical school		32 (7.1%)	6 (3%)	12 (9%)	14 (11.7%)
Some college		95 (21%)	32 (16.1%)	34 (25.6%)	29 (24.2%)
College graduate		136 (30.1%)	71 (35.7%)	34 (25.6%)	31 (25.8%)
Postgraduate		96 (21.2%)	56 (28.1%)	27 (20.3%)	13 (10.8%)

^a^Education level was not a question asked in our qualitative protocol

As a convergent design, we conducted an integrated analysis, evaluating qualitative and quantitative data iteratively. Thus, results from the qualitative and quantitative phases are integrated throughout the four categories we identified in response to the research question: enculturated weights and stereotypes, life stressors, protective factors, and health impact.

### Culture Weighs Them Down: Enculturated Weights and Stereotypes

Women described *enculturated weights and stereotypes* that negatively influence their health and their ability to withstand stress. Women reflected on the expectation both within and from outside their culture that Black women should be strong, self-reliant, and able to handle adversity without showing vulnerability. They described a deep sense of obligation to live up to these ideals, especially in the context of their identities as educated Black women or single mothers. The expectation to always “keep it together” in both personal and professional spheres resulted in the suppression of emotional needs, further compounding their stress. For instance, women felt compelled to take on numerous responsibilities within their families and communities, often without receiving support in return. Sara (age 27–39) extended the metaphor, “So you let your own health lack to save the world, even though you may be killing yourself… Having on the cape means doing everything—taking care of this person, being a mom, being a strong Black woman—until you’re so strong that you’ll do what you have to do, even with a broken arm and two broken legs.”

The cultural expectation to suppress emotions and pain weighed women down. Sunny (age 18–26) summarized, “growing up…nobody really got to express their emotions…if you did, you just getting yelled at and you’re not supposed to, so it kind of translates now into adulthood, … nobody talk about their emotions with each other.” Women attributed this behavior to their racial culture. Jane (age 27–39) reflected on the pressure for Black women to carry burdens while limiting their emotional responses: “You have to be emotionless when times are hard… I feel like it’s expected of Black women to be like that.”

A culturally linked mental health stigma intensified this weight. Women described that, as a culture, Black individuals often downplay, overlook, or ignore mental health concerns. Girty (age 27–39) reflected, “I think that really comes from our ancestors…Everybody throws everything up under the rug…as a Black child, you can’t go to your parents and say, ‘hey, I’m depressed’…they’re going to tell you… ‘go get over it, go outside, go play.’” Women also described the expectation to persevere through physical pain. Tiana (age 27–39) shared, “I’ve went almost 10 years of my life with lower quadrant abdominal pain, heavy menstrual cycles, not knowing that I had endometriosis, afraid to say, ‘I can’t go in to work today’ or ‘I’m tired,’ …my mom was like, ‘that is the norm, don’t fuss about it, deal with it.’”.

These cultural norms were tangled with a cultural expectation to be self-reliant. Women expressed how limited expectations for strong support or supportive partners have become normalized, reflecting a broader cultural experience. Nicole (age 40–49) explained, “…I just feel like Black women don’t have the support like the other races do… they don’t have nobody that they can depend on, and I just think that other races do, especially Caucasian…” HINTS6 data supported interviewees’ normative beliefs. Black women were more likely to be single and never married (χ ^2^(4),* N* = 452) = 47.47,* p* < 0.001) than Hispanic women or White women.

Women described the tension between their true sense of self and the stereotypes projected onto them. Women described how words like “angry,” “bitter,” or “loud” stereotype them. Women emphasized how their emotions are frequently misinterpreted, reflecting a broader misunderstanding of Black women’s experiences and feelings. Dispatcher (age 40–49) said, “…people usually stereotype me…if you verbally express a boundary, now you’re being rude or loud.” Women expressed that they felt a need to resist assumed stereotypes. Kay (age 40–49) emphasized, “We’re always having to watch our tone, our facial expression, watching what we say…that gets hard and stressful sometimes.”

To better understand the potential uniqueness of the experiences of Black women, we compared the racial/ethnic groups from the HINTS6 data on ethnic belonging. Black women reported a significantly stronger sense of ethnic belonging than both White women and Hispanic women (*F*(2,446) = 29.17,* p* < 0.001). This quantitative difference highlights the strong influence that Black women experience from within their culture.

### “Stress About that Every Day”: Life Stressors

Black women described a variety of life stressors related to their relationships, their workplaces, and their communities. Relationally, *family caregiving* generated stress. Women described the stress of “doing everything on my own” and linked it to their physical health. Monique (age 27–39) explained, “… [child’s father] will say…’you can do it, you’ve been doing it.’ I have been doing it for 12 years, but that doesn’t mean I want to do it…I’m just making it work.” She connected this stressful expectation to race, saying, “That’s something that actually makes me upset because, being a mother, especially a Black mother, you’re just automatically supposed to do…everything.” For some women, the stress of family prompted them to withdraw from family situations. Ashley (age 27–39) explained, “I try to stay secluded…I don’t feel like other people understand.” Withdrawal was critical in stressful situations. Jane Doe (age 18–26) said, “… mentally and physically, I had to remove myself. I want [my family] to know that I’m still there for them.”

Women described how they prioritized family members over themselves. Pearl (age 27–39) summarized, “As Black women, we’re so accustomed to being stressed that we overlook our own mental health…We have so much on our plates and not enough time, and we put everybody else before us. We ignore our own health overall.” Women specifically described stressors associated with raising Black children. Sara (age 27–39) explained, “…because one wrong move, if [my son] talks loud, if he bumps into a White girl at school…what are they gonna see…it’s a constant fear.” The stress of Black motherhood was amplified for single parents. Coco (age 27–39) described, “…if I was a White woman, I would not be in this situation at all…I never thought I would be a single mother, but I am… all the pressure falls on you…I actually stress about that every day.”

The *workplace* introduced multiple stressors. Women described a caste system, which Black women are treated as inferior. Pearl (age 27–39) said, “…we’re a double minority anyway, we’re Black, and then we’re women, so we’re already leveled under, so we have to do so much more…so I still had odds against me just to get on an even playing field.” Women reported that they cannot reach their full potential due to their identity. Monique (age 27–39) recounted, “There was a team lead position that came available that I knew I was very qualified for, but I was passed over for a White male…I got a lot of feedback from people outside of management that definitely agreed with me that that role was better suited for me, but of course, as a Black woman, I wouldn’t be at the top of the list for those type of promotions.”

Pressures to *maintain a “professional” appearance* added stress. Women described wearing looser clothing or refraining from wearing their hair in its natural state to fit professional standards. Tiana (age 27–39) recounted how her mother had trained her to believe that straight hair was professional. Then, people in the workforce reinforced that belief. Tiana said, “…being a healthcare provider…I can’t wear my natural hair. I have to make sure it’s straight or I have to put on a wig…[patients] see my white coat or they see who I am, and they say, ‘are you my provider…you can’t be this because your hair is kinky or your skin doesn’t look like mine, so you have to be someone in janitorial service…’” Eventually, Tiana was diagnosed with “a form of alopecia that’s more prevalent in the African American community.” For Black women, the relationship between sociocultural pressure to look a certain way and the resulting stress caused health problems.

*Limited housing choice* was a source of stress. Women described the stress associated with the costs of housing. For women who relied on subsidized housing programs, the complexity and uncertainty of the administrative processes created stress. Safety was also linked to limited housing choice. Pearl (age 27–39) shared, “I actually live in low-income housing…there was a drive-by shooting. Two bullets went through my window…two inches from where I would have been sitting…so, it’s definitely not safe.” She described her living situation as “very much stressful.”

Here, we tested group differences in feelings about household income since it was connected to housing choice. Black women reported worse feelings about household income than non-Hispanic White and Hispanic women, *F*(2,442) = 3.19, *p* < 0.05. A post hoc test showed that the statistically significant difference was between Black and White women. See Table 1.

Lastly, the *healthcare system experiences* created stress for women, including medical discrimination and illness invalidation. Women expected that if they were another race, they would receive better care. Susan (age 40–49) shared, “I feel like they don’t hear me because of the color of my skin…I’m crying out for help, it’s still like they’re not hearing me [*tearful*].” This discrimination regularly took the form of illness invalidation. When going through the diagnostic process for a pituitary tumor, Crystal (age 27–39) encountered a doctor who was “a White woman and she told me that I was just having frontal headaches and it’ll be all right… If you don’t advocate for yourself, they’ll run over you…”.

Quantitatively, we compared women’s evaluation of patient-centered communication as reported in HINTS6. Black women report lower patient-centered communication than non-Hispanic White women (n.s.) but higher than Hispanic women (n.s.).

### Protective Factors

Black women described four protective factors that protect them through stressors: positive outlook, faith-related beliefs, consistent practices, and social support. An overall *positive outlook* protected women when they encountered stress. Rita (age 40–49) shared, “My kids tell me that all the time, ‘we can’t believe that you just sit around when stuff be going wrong and you just be like it’s gonna be alright’…I grew up like that, so I guess that’s why it’s stuck in me.” *Faith-related beliefs* also helped women respond to stressors. Twin (age 27–39) said, “Some things honestly I don’t think I would have gotten through if it wasn’t for my faith, just being a believer…it’s not something that I have to waiver with, it’s something that I know for sure… I think it’s very important to have that and to be able to stand on that.”

In the quantitative data, Black women were distinguishable from other women by meaning and purpose in life, a potential protective factor that reflects positive outlook and faith-related beliefs. Black women reported greater meaning and purpose than both White and Hispanic women, *F*(2,445) = 4.81, *p* < 0.01. A post hoc test showed that the statistically significant difference was between Black and White women. See Table 1.

Separately, *consistent practices* prepared them to handle everyday life stressors or stressors that they may experience more frequently. Consistent practices were behaviors women regularly engaged in, including exercise and hobbies. Some practices were religious-oriented (reading the bible, praying, attending church/bible study), but their active nature distinguished them from faith-related beliefs. Women also described how they utilized these practices in response to unexpected stressful life events. Sunny 2 (age 18–26) described her responses, “I draw, I sing, I write…my coping mechanisms that I really go to.” Health-promotive behaviors, such as eating cleaner, also helped reduce stress. Stacy (age 40–49) described how she changed her lifestyle after her mom dealt with significant health issues, “I run. I manage my stress. I do not eat as healthy as I could, but I sleep well, I make sure I don’t drink too much.”

Lastly, women described the protective nature of *social support*. Women described people who offer help with the interviewee’s responsibilities or other activities that would prevent stress. For some women, social support was familial. Sara (age 27–39) explained, “…[my husband] cooks, he cleans, he folds clothes, he washes clothes. He helps do everything which is awesome…” Other women described extra-familial support. Gigi (age 40–49) said, “I have what I call my squad, and five people that any given time, I can call or text and they will be right there, or I can tell them anything and I know it won’t go any further.”

Social support was critical for women’s response to stressors. Women communicated with specific family members about stressful events in hopes of resolving tension. Tiana (age 27–39) recalled, “So I called a family meeting ‘cause it was weighing on my heart, and I knew that I’m entering this new phase in my life where my peace is very important, so let me say what’s on my mind and my heart.” Women also went to counseling. Kenya (age 27–39) said, “I felt like it was stuff that I had not dealt with in my mom’s passing, trying to keep my family together, lack of support, and it just became overwhelming, … so that’s when I went to talk to somebody.”

Quantitatively, we tested for group differences on social isolation, which can be conceptualized as the absence of social support. However, an ANOVA demonstrated no group differences on the variable. See Table 1.

### Model of How Sociocultural Identities and Experiences of Black Women Impact Their Health

**Fig. 2:**
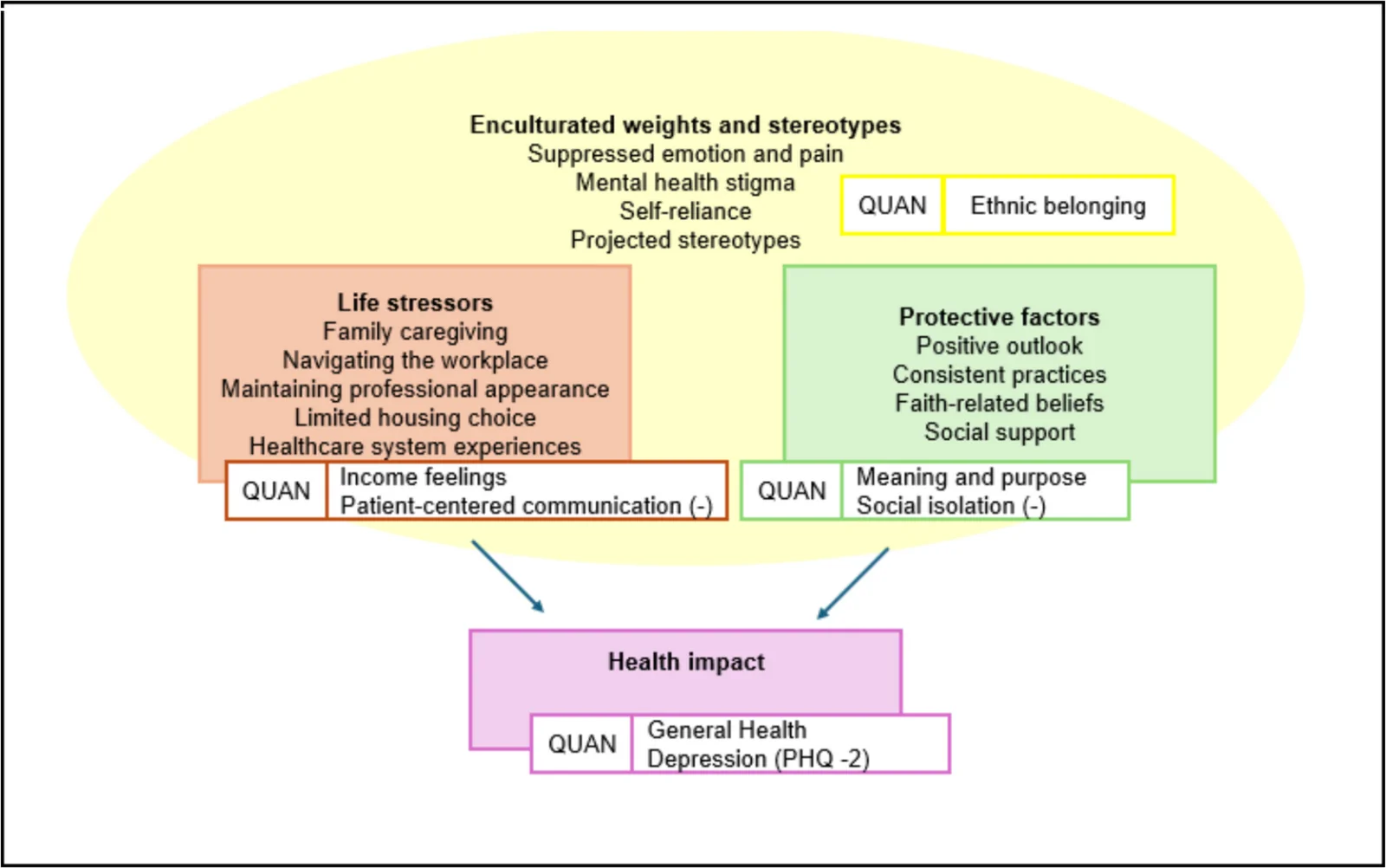
Model of how sociocultural identities and experiences of Black women impact their health

The resulting model illustrated how sociocultural factors, life stressors, and protective factors impacted Black women’s physical and mental health. See Fig. 2. Monique (age 27–39) explained, “I have to keep on a brave face to make sure my kids don’t see me down…It’s stressful because holding in how you feel can cause tension on your body, and it causes more stress, because you’re stressing but you’re not saying anything…” Women experienced life stressors and protective factors within the context of their culture. Enculturated weights and stereotypes amplified stressors and limited protective factors. Nicole (age 40–49) described how stress “[takes] a physical toll on Black women.” Women also described specific impacts such as hair loss, sleeplessness, weight gain, and even shingles due to the stress in their lives.

To quantitatively explore the effect of identity, stressors, and protective factors on health, we tested the effect of race/ethnicity, marital status, income feelings, patient-centered communication, meaning and purpose, and social isolation on two health outcomes in HINTS6: general health and Patient Health Questionnaire (PHQ) 2.

In a full factorial analysis of covariance (ANCOVA), race/ethnicity and marital status did not have statistically significant main or interaction effects on general health. However, income feelings (*p* < 0.001), isolation (*p* < 0.05), and meaning and purpose (*p* < 0.01) were associated with general health. Women who reported worse feelings about income, greater social isolation, or less meaning and purpose also reported worse general health.

**Fig. 3:**
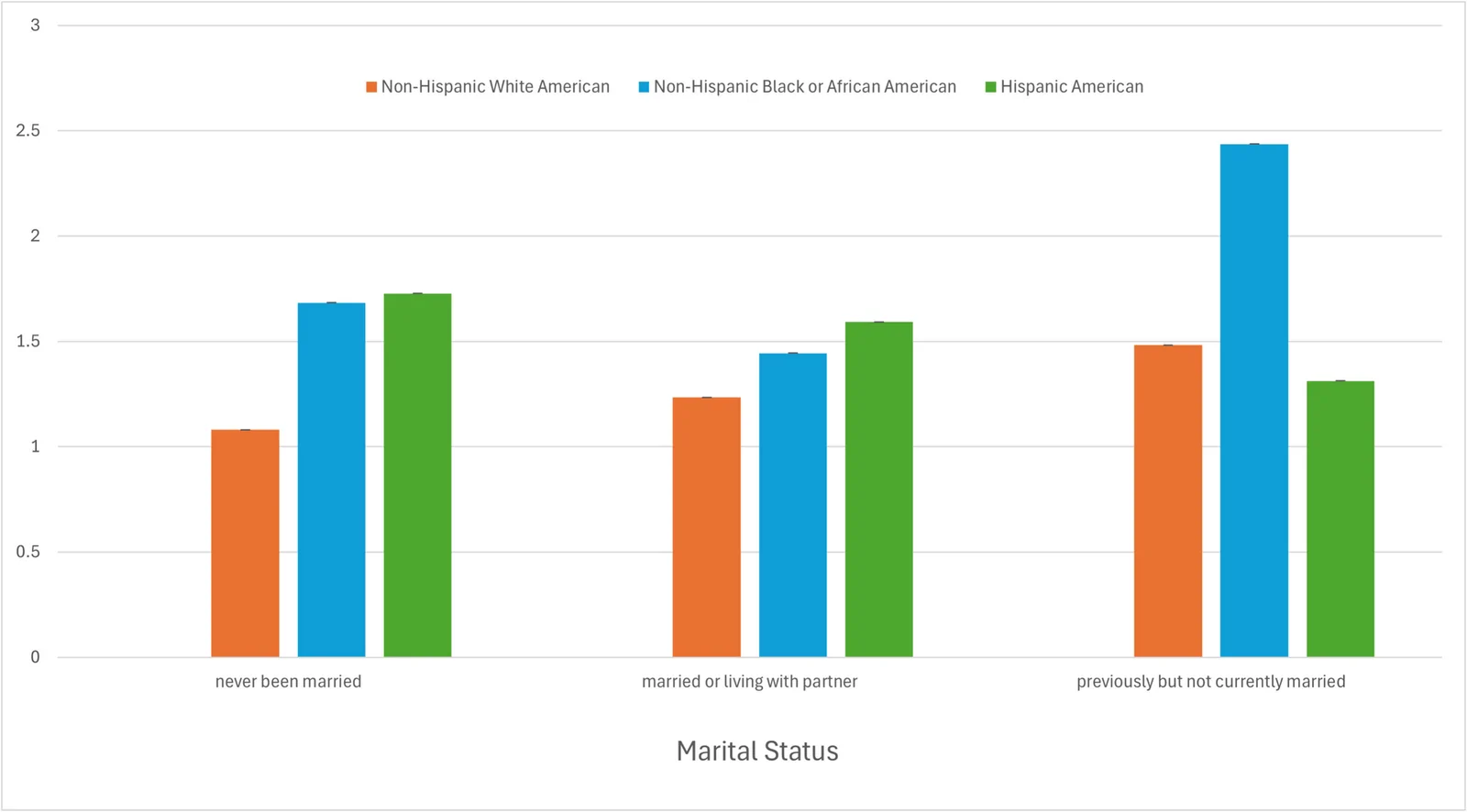
Analysis of covariance* testing the effects of race/ethnicity and marital status on depression, as measured by the Patient Health Questionnaire (PHQ) 2. *Full model included race/ethnicity, marital status, meaning and purpose, income feelings, patient-centered communication, meaning and purpose, and social isolation, Patient Health Questionnaire (PHQ) 2

In a second ANCOVA on the PHQ-2, the test demonstrated a significant main effect for race/ethnicity, *F*(4,428) = 5.166, *p* < 0.01. See Fig. 3 for estimated marginal means. Black women who were divorced or separated had the highest mean, 2.69 on the PHQ-2. This score approaches clinical significance; a score of 3 or higher indicates that major depressive disorder is likely. Income feelings (*p* < 0.01), meaning and purpose (*p* < 0.001), and social isolation (*p* < 0.001) also had statistically significant effects. Women who reported worse feelings about income, greater social isolation, or less meaning and purpose also scored higher with depressive indicators.

## Discussion

This study reveals how Black women’s sociocultural identities shape their experiences with stress and significantly impact their health. These identities are not just labels but reflect deeply ingrained roles that influence both their internal expectations and external experiences. Although these roles can provide a sense of pride and purpose, they also contribute to an ongoing burden of stress, particularly when women feel pressured to uphold these identities in the face of systemic barriers and discrimination. Women expressed a strong sense of responsibility to be self-reliant and capable, particularly in the context of their roles as caregivers, professionals, and community leaders. Findings align with prior studies showing that Black women disproportionately engage in “emotional labor” and self-silencing to conform to expectations of resilience, which can ultimately compromise their well-being [53–57].

Participants’ narratives pointed to increased emotional and physiological strain consistent with literature on gendered racism and stress embodiment, including elevated rates of anxiety, depression, and chronic health risks among Black women [58–60]. These findings align with growing research on allostatic load, which links cumulative exposure to discrimination with physiological dysregulation across the cardiovascular, metabolic, and immune systems [9, 61–64]. In particular, high-effort coping strategies such as John Henryism and caregiving under chronic strain have been associated with physiological stress responses that increase vulnerability to inflammation and chronic illness, especially among African American populations [65–68]. Interviewees’ reflections on medical mistrust, housing instability, and workplace and healthcare discrimination add richness to prior work identifying structural stressors as critical drivers of health disparities [4, 7, 8]. The intersectionality of race and gender emerged as a key determinant of participants’ stress experiences. Black women navigated the compounded effects of being both Black and a woman. Intersectionality provides a useful framework for understanding how these overlapping identities expose Black women to unique stressors that differ from those experienced by Black men or White women [69]. These findings extend the superwoman schema (SWS), a model which describes a race-gender role orientation adopted by Black women to withstand the oppressive sociohistorical and sociopolitical context of gendered racism [56, 70]. Our findings are consistent with the literature that positions the SWS as both a culturally rooted strength and a risk factor for psychological distress, particularly when combined with limited social support or economic strain [71, 72].

The mixed-methods approach strengthened our ability to understand this intersectionality. Although the quantitative data could not fully capture the complexity of identity-based stress, it offered important contextual insight. Specifically, constructs like income dissatisfaction, meaning and purpose, and depressive symptoms helped situate participants’ narratives within broader population-level trends. For example, the significantly higher PHQ-2 scores among divorced or separated Black women aligned with qualitative themes of emotional exhaustion, grief, and invisibility. This reinforces findings from the Black Women’s Health Study and other population-based cohorts that show marital status and social support as key moderators of mental health risk for Black women [73–76]. Similarly, Black women’s stronger sense of ethnic belonging and higher reported sense of purpose reflect the protective nature of racial identity and spirituality documented in prior studies [77, 78].

Despite the diversity of women’s life experiences within the qualitative sample, their narratives reflected a strong sense of shared identity that cut across other demographic differences. These findings spotlight the importance of ascribed identity [79]—how others see and name their Blackness and womanhood in ways that overshadow and constrain Black women’s identities as professionals, caregivers, or individuals. To support meaningful integration of these perspectives, the quantitative subsample was matched to the qualitative group in terms of age, region, and household characteristics, ensuring consistency across strands. Our conceptual model (Fig. 2) illustrates how sociocultural identity, chronic stressors, and protective factors coalesce to shape health outcomes. Developed through an integrated analysis of both qualitative and quantitative data, the model offers a framework to understand how internalized expectations can amplify stress and inhibit support-seeking behavior.

Although this study did not assess disease-specific outcomes, the chronic stressors described by participants—emotional suppression, limited support, racial discrimination, and high-effort coping—align with biological pathways linked to breast cancer disparities among Black women. Studies show that elevated allostatic load, a marker of cumulative physiological stress, is associated with more aggressive tumor features and poorer breast cancer outcomes in Black women [80, 81]. These findings support frameworks such as “weathering” and “society-to-cells-to-outcomes,” which describe how chronic exposure to structural racism and social stressors results in neuroendocrine and immune dysregulation [82, 83]. Elevated stress has been linked to higher breast cancer mortality, independent of socioeconomic and tumor-specific factors, suggesting that identity-related stress may play a mechanistic role in outcome disparities [84]. This body of evidence underscores the importance of addressing social and structural stressors in cancer prevention research—particularly for Black women, whose lived experiences may accelerate disease risk through biologically embedded stress responses [85, 86].

Several limitations should be considered when interpreting results. First, all interviewees lived in the Southeastern USA, and those who voiced faith-related beliefs shared a similar faith. Although we aligned the quantitative subsample with our qualitative group on age, region, and racial identity to support integration, the national survey data were not designed to measure identity-based stress explicitly. As such, the quantitative findings serve as contextual support rather than direct confirmation of qualitative themes. Additionally, although our interdisciplinary research team includes Black and non-Black women trained in qualitative and mixed-methods research, we acknowledge that our interpretations were shaped by our positionalities.

Future work should build on this study by expanding to additional geographic regions, incorporating longitudinal data, and developing new quantitative instruments that better capture identity-related stress and sociocultural coping, particularly in relation to chronic disease and mental health outcomes. Future research should use longitudinal designs to assess how identity-based stress accumulates across the life course and should explore culturally embedded protective factors that may buffer these effects. Mixed-methods designs remain essential in this effort, offering both breadth and depth in addressing persistent racial and gender health inequities.

## Conclusion

This study explored two primary research questions: (1) How do Black women describe their sociocultural identity and stressors within their families, workplaces, and communities? (2) How are these identities and stressors connected to physical and mental health outcomes? Findings from both qualitative and quantitative strands of the study addressed these questions by illustrating how culturally embedded identities—particularly expectations around strength, caregiving, and emotional control—contribute to mental health strain and, in some cases, physical symptoms consistent with chronic stress exposure. These findings suggest that sociocultural identity plays a central role in shaping stress experiences among Black women and should be explicitly integrated into health equity efforts. Health interventions must address both the internalized expectations and the external structural barriers that drive cumulative stress. Supporting Black women’s health and well-being requires approaches that are culturally grounded, intersectional, and responsive to both psychological and physiological needs.

## Data Availability

The quantitative data analyzed in this study are publicly available from the Health Information National Trends Survey (HINTS) repository (https://hints.cancer.gov/). The qualitative interview data generated and analyzed during the current study are not publicly available due to confidentiality agreements with participants but may be available from the corresponding author on reasonable request, subject to institutional and ethical approval.

## References

[1] Chinn JJ, Martin IK, Redmond N. Health equity among Black women in the United States. J Womens Health. 2021;30(2):212–9. 10.1089/jwh.2020.8868 PMC8020496 33237831

[2] Palmer JR, Cozier YC, Rosenberg L. Research on health disparities: strategies and findings from the Black women’s health study. Am J Epidemiol. 2022;192(11):1806–10. 10.1093/aje/kwac022. PMC11004793 35136921

[3] Shoemaker ML, White MC, Wu M, Weir HK, Romieu I. Differences in breast cancer incidence among young women aged 20–49 years by stage and tumor characteristics, age, race, and ethnicity, 2004–2013. Breast Cancer Res Treat. 2018;169(3):595–606. 10.1007/s10549-018-4699-9. PMC5955792 29445940

[4] Richardson LC. Patterns and trends in age-specific Black-white differences in breast cancer incidence and mortality–United States, 1999–2014. MMWR Morb Mortal Wkly Rep. 2016. 10.15585/mmwr.mm6540a1. 27736827

[5] Newman LA. Breast cancer disparities: high-risk breast cancer and African ancestry. Surg Oncol Clin N Am. 2014;23(3):579–92. 10.1016/j.soc.2014.03.014 24882352

[6] Dunn BK, Agurs-Collins T, Browne D, Lubet R, Johnson KA. Health disparities in breast cancer: biology meets socioeconomic status. Breast Cancer Res Treat. 2010;121:281–92. 10.1007/s10549-010-0827-x 20437200

[7] Giaquinto AN, et al. Breast cancer statistics, 2022. CA Cancer J Clin. 2022;72(6):524–41. 10.3322/caac.21754. 36190501

[8] Jatoi I, Sung H, Jemal A. The emergence of the racial disparity in U.S. breast-cancer mortality. N Engl J Med. 2022;386(25):2349–52. 10.1056/NEJMp2200244. 35713541

[9] Duru OK, Harawa NT, Kermah D, Norris KC. Allostatic load burden and racial disparities in mortality. J Natl Med Assoc. 2012;104(1–2):89–95. 10.1016/s0027-9684(15)30120-6. PMC3417124 22708252

[10] Shchaslyvyi AY, Antonenko SV, Telegeev GD. Comprehensive review of chronic stress pathways and the efficacy of behavioral stress reduction programs (BSRPs) in managing diseases. Int J Environ Res Public Health. 2024. 10.3390/ijerph21081077. (**(in eng)**). PMC11353953 39200687

[11] A. Mariotti, “The effects of chronic stress on health: new insights into the molecular mechanisms of brain-body communication,” (in eng), *Future Sci OA,* vol. 1, no. 3, p. Fso23, Nov 2015, 10.4155/fso.15.21. PMC5137920 28031896

[12] Roberts BL, Karatsoreos IN. Brain-body responses to chronic stress: a brief review. Fac Rev. 2021;10:83. 10.12703/r/10-83. PMC8725649 35028648

[13] Yaribeygi H, Panahi Y, Sahraei H, Johnston TP, Sahebkar A. “The impact of stress on body function: a review”, (in eng). Excli j. 2017;16:1057–72. 10.17179/excli2017-480. PMC5579396 28900385

[14] Moreno-Smith M, Lutgendorf SK, Sood AK. Impact of stress on cancer metastasis. Future Oncol. 2010;6(12):1863–81. 10.2217/fon.10.142. PMC3037818 21142861

[15] P. A. Volden and S. D. Conzen, “The influence of glucocorticoid signaling on tumor progression,” (in eng), *Brain Behav Immun,* vol. 30 Suppl, no. 0, pp. S26–31, Mar 2013, 10.1016/j.bbi.2012.10.022. PMC3987853 23164950

[16] Dai AQS, et al. Chronic stress promotes cancer development. Front Oncol. 2020. 10.3389/fonc.2020.01492. PMC7466429 32974180

[17] A. Nishida and A. Andoh, “The role of inflammation in cancer: mechanisms of tumor initiation, progression, and metastasis,” *Cells,* vol. 14, no. 7, p. 488, 2025. [Online]. Available: https://www.mdpi.com/2073-4409/14/7/488. 10.3390/cells14070488 PMC11987742 40214442

[18] Liu Y, Tian S, Ning B, Huang T, Li Y, Wei Y. Stress and cancer: the mechanisms of immune dysregulation and management. Front Immunol. 2022. 10.3389/fimmu.2022.1032294. (**(in English)**). PMC9579304 36275706

[19] Yan J, et al. Chronic stress in solid tumor development: from mechanisms to interventions. J Biomed Sci. 2023;30(1):8. 10.1186/s12929-023-00903-9. PMC9883141 36707854

[20] Kishi S, et al. “Cumulative blood pressure in early adulthood and cardiac dysfunction in middle age: the CARDIA Study”, (in eng). J Am Coll Cardiol. 2015;65(25):2679–87. 10.1016/j.jacc.2015.04.042. 26112189

[21] Winpenny EM, Howe LD, van Sluijs EMF, Hardy R, Tilling K. Early adulthood socioeconomic trajectories contribute to inequalities in adult cardiovascular health, independently of childhood and adulthood socioeconomic position. J Epidemiol Community Health. 2021;75(12):1172–80. 10.1136/jech-2021-216611. PMC8588297 34362821

[22] Sabia S, Elbaz A, Rouveau N, Brunner EJ, Kivimaki M, Singh-Manoux A. Cumulative associations between midlife health behaviors and physical functioning in early old age: a 17-year prospective cohort study. J Am Geriatr Soc. 2014;62(10):1860–8. 10.1111/jgs.13071. PMC4206608 25283337

[23] Bailey ZD, Krieger N, Agénor M, Graves J, Linos N, Bassett MT. Structural racism and health inequities in the USA: evidence and interventions. Lancet. 2017;389(10077):1453–63. 10.1016/S0140-6736(17)30569-X. 28402827

[24] Krieger N, Jahn JL, Waterman PD. Jim Crow and estrogen-receptor-negative breast cancer: US-born Black and white non-Hispanic women, 1992–2012. Cancer Causes Control. 2017;28(1):49–59. 10.1007/s10552-016-0834-2. (**(in eng)**). 27988896

[25] Gee GC, Ford CL. Structural racism and health inequities: old issues, new directions. Du Bois Rev Soc Sci Res Race. 2011;8(1):115–32. 10.1017/S1742058X11000130. PMC4306458 25632292

[26] Williams DR, Lawrence JA, Davis BA, Vu C. Understanding how discrimination can affect health. Health Serv Res. 2019;54(Suppl 2):1374–88. 10.1111/1475-6773.13222. PMC6864381 31663121

[27] Gee GC, Payne-Sturges DC. “Environmental health disparities: a framework integrating psychosocial and environmental concepts”, (in eng). Environ Health Perspect. 2004;112(17):1645–53. 10.1289/ehp.7074. PMC1253653 15579407

[28] Williams DR, Mohammed SA. Racism and health i: pathways and scientific evidence. Am Behav Sci. 2013;57(8):1152–73. 10.1177/0002764213487340. PMC3863357 24347666

[29] Zenk SN, Lachance LL, Schulz AJ, Mentz G, Kannan S, Ridella W. Neighborhood retail food environment and fruit and vegetable intake in a multiethnic urban population. Am J Health Promot. 2009;23(4):255–64. 10.4278/ajhp.071204127. PMC3305995 19288847

[30] Alcaraz KI, Wiedt TL, Daniels EC, Yabroff KR, Guerra CE, Wender RC. Understanding and addressing social determinants to advance cancer health equity in the United States: a blueprint for practice, research, and policy. CA Cancer J Clin. 2020;70(1):31–46. 10.3322/caac.21586. 31661164

[31] M. Paul, Z. Khaing, and W. and Darity, “Returns in the labor market: a nuanced view of penalties at the intersection of race and gender in the US,” *Feminist Economics,* vol. 28, no. 2, pp. 1–31, 2022/04/03 2022, 10.1080/13545701.2022.2042472.

[32] Dill J, Duffy M. Structural racism and Black women’s employment in the US health care sector. Health Aff (Millwood). 2022;41(2):265–72. 10.1377/hlthaff.2021.01400. PMC9281878 35130061

[33] R. Mehra *et al.*, ““‘Oh gosh, why go?’ cause they are going to look at me and not hire”: intersectional experiences of Black women navigating employment during pregnancy and parenting,” (in eng), *BMC Pregnancy Childbirth,* vol. 23, no. 1, p. 17, Jan 10 2023, 10.1186/s12884-022-05268-9. PMC9830615 36627577

[34] Shaw E, Mason CN, Lacarte V, Jauregui E. Holding up half the sky: mothers as workers, primary caregivers, & breadwinners during COVID-19. Washington, DC: Institute for Women’s Policy Research; 2020.

[35] Fabius CD, Wolff JL, Kasper JD. Race differences in characteristics and experiences of Black and White caregivers of older Americans. Gerontologist. 2020;60(7):1244–53. 10.1093/geront/gnaa042. PMC7491434 32400881

[36] D. R. Williams and L. A. Cooper, “Reducing racial inequities in health: using what we already know to take action,” (in eng), *Int J Environ Res Public Health,* vol. 16, no. 4, Feb 19 2019, 10.3390/ijerph16040606. PMC6406315 30791452

[37] J. A. Lewis, R. Mendenhall, S. A. Harwood, and M. Browne Huntt, “Coping with gendered racial microaggressions among Black women college students,” *Journal of African American Studies,* vol. 17, no. 1, pp. 51–73, 2013/03/01 2013, 10.1007/s12111-012-9219-0.

[38] Allen AM, et al. Racial discrimination, the superwoman schema, and allostatic load: exploring an integrative stress-coping model among African American women. Ann N Y Acad Sci. 2019;1457(Dec):104–27. 10.1111/nyas.14188. PMC6904516 31403707

[39] Chambers BD, et al. Exposures to structural racism and racial discrimination among pregnant and early post-partum Black women living in Oakland, California. Stress Health. 2020;36(Apr):213–9. 10.1002/smi.2922. PMC7269549 31919987

[40] Geronimus AT, Hicken M, Keene D, Bound J. ‘Weathering’ and age patterns of allostatic load scores among Blacks and whites in the United States. Am J Public Health. 2006;96(5):826–33. 10.2105/ajph.2004.060749. PMC1470581 16380565

[41] Glaser BG, Strauss AL. The discovery of grounded research: strategies for qualitative research. New York: Aldine De Gruyter; 1967.

[42] Charmaz K. Constructing grounded theory: a practical guide. London, England: Sage; 2006.

[43] Glaser BG. Conceptualization: on theory and theorizing using grounded theory. Int J Qual Methods. 2002;1:1–31.

[44] C. Conlon, V. Timonen, C. Elliott-O’Dare, S. O’Keeffe, and G. Foley, “Confused about theoretical sampling? Engaging theoretical sampling in diverse grounded theory studies,” (in eng), *Qual Health Res,* vol. 30, no. 6, pp. 947–959, May 2020, 10.1177/1049732319899139. 31959073

[45] Braun V, Clarke V. To saturate or not to saturate? Questioning data saturation as a useful concept for thematic analysis and sample-size rationales. Qual Res Sport Exerc Health. 2021;13(2):201–16.

[46] S. B. Thomson, “Sample size and grounded theory,” *Journal of Administration and Governance,* vol. 5, no. 1, 2011.

[47] Wutich A, Beresford M, Bernard HR. Sample sizes for 10 types of qualitative data analysis: an integrative review, empirical guidance, and next steps. Int J Qual Methods. 2024;23:16094069241296206.

[48] Itzik L, Walsh SD. Giving them a choice: qualitative research participants chosen pseudonyms as a reflection of self-identity. J Cross-Cult Psychol. 2023;54(6–7):705–21. 10.1177/00220221231193146.

[49] Maietta R, Mihas P, Swartout K, Petruzzelli J, Hamilton A. Sort and sift, think and shift: let the data be your guide an applied approach to working with, learning from, and privileging qualitative data. Qual Rep. 2021. 10.46743/2160-3715/2021.5013.

[50] Morse JM, Barrett M, Mayan M, Olson K, Spiers J. Verification strategies for establishing reliability and validity in qualitative research. Int J Qual Methods. 2002;1(2):13–22.

[51] Hesse BW, Moser RP, Rutten LJF, Kreps GL. The health information national trends survey: research from the baseline. J Health Commun. 2006;11(Suppl 1):vii–xvi. 10.1080/10810730600692553. 16641070

[52] *IBM SPSS Statistics*. (2021). IBM Corp.

[53] Watson-Singleton NN, Womack VY, Holder-Dixon AR, Black AR. Racism’s (un)worthiness trap: the mediating roles of self-compassion and self-coldness in the link between racism and distress in African Americans. Cultur Divers Ethnic Minor Psychol. 2022;28(4):557–66. 10.1037/cdp0000398. 34726424

[54] J. A. Abrams, A. Hill, and M. Maxwell, “Underneath the mask of the strong Black woman schema: disentangling influences of strength and self-silencing on depressive symptoms among U.S. Black women,” (in eng), *Sex Roles,* vol. 80, no. 9–10, pp. 517–526, May 2019, 10.1007/s11199-018-0956-y. PMC6510490 31086431

[55] Abrams JA, Maxwell M, Pope M, Belgrave FZ. Carrying the world with the grace of a lady and the grit of a warrior:deepening our understanding of the “strong Black woman” schema. Psychol Women Q. 2014;38(4):503–18. 10.1177/0361684314541418.

[56] Woods-Giscombé CL. Superwoman schema: African American women’s views on stress, strength, and health. Qual Health Res. 2010;20(5):668–83. 10.1177/1049732310361892. PMC3072704 20154298

[57] Erving CL, et al. Black women as superwomen? The mental health effects of superwoman schema, socioeconomic status, and financial strain. Soc Probl. 2024;72(2):759–82. 10.1093/socpro/spae007. PMC12169892 40530389

[58] M. M. Vance, J. Wade, G. Gowdy, and H. Dillon, “Racialized gender stress and mental health among Black women: a test of the Vance-Wade Intersectional Suicide Risk Model with longitudinal data,” *Journal of Racial and Ethnic Health Disparities,* 2024/09/30 2024, 10.1007/s40615-024-02076-z. 39349902

[59] Nelson T, Brown MJ, Garcia-Rodriguez I, Moreno O. Gendered racism, anxiety, and depression: the mediating roles of gendered racialized stress and social isolation. Ethn Health. 2023;28(1):12–28. 10.1080/13557858.2021.2002826. 34781804

[60] Robinson MN, Erving CL, Tobin CST. Are distressed Black women also depressed? Implications for a mental health paradox. J Racial Ethn Health Disparities. 2023;10(3):1280–92. 10.1007/s40615-022-01313-7. PMC9652478 35556224

[61] Moore JX, Bevel MS, Aslibekyan S, Akinyemiju T. Temporal changes in allostatic load patterns by age, race/ethnicity, and gender among the US adult population; 1988–2018. Prev Med. 2021;147:106483. 10.1016/j.ypmed.2021.106483. PMC8826516 33640399

[62] Perry BL, Harp KL, Oser CB. Racial and gender discrimination in the stress process: implications for African American women’s health and well-being. Sociol Perspect. 2013;56(1):25–48. PMC3783344 24077024

[63] Upchurch DM, et al. A longitudinal investigation of race, socioeconomic status, and psychosocial mediators of allostatic load in midlife women: findings from the study of women’s health across the nation. Psychosom Med. 2015;77(May):402–12. 10.1097/psy.0000000000000175. PMC4431938 25886828

[64] Moore JX, Bevel MS, Aslibekyan S, Akinyemiju T. Temporal changes in allostatic load patterns by age, race/ethnicity, and gender among the US adult population; 1988–2018. Prev Med. 2021;147:106483. 10.1016/j.ypmed.2021.106483 PMC8826516 33640399

[65] G. G. Bennett *et al.*, “Stress, coping, and health outcomes among African-Americans: a review of the John Henryism hypothesis,” *Psychology & Health,* vol. 19, no. 3, pp. 369–383, 2004/06/01 2004, 10.1080/0887044042000193505.

[66] R. Clark, N. B. Anderson, V. R. Clark, and D. R. Williams, “Racism as a stressor for African Americans. A biopsychosocial model,” (in eng), *Am Psychol,* vol. 54, no. 10, pp. 805–16, Oct 1999, 10.1037//0003-066x.54.10.805. 10540593

[67] Clark R, Adams JH, Clark VR. Effects of John Henryism and anger-coping on mean arterial pressure changes in African American women. Int J Behav Med. 2001;8:270–81.

[68] Woods-Giscombé CL, Lobel M. “Race and gender matter: a multidimensional approach to conceptualizing and measuring stress in African American women”, (in eng). Cultur Divers Ethnic Minor Psychol. 2008;14(3):173–82. 10.1037/1099-9809.14.3.173. PMC2553624 18624581

[69] Crenshaw K. Mapping the margins: intersectionality, identity politics, and violence against women of color. Stanford Law Rev. 1991;43(6):1241–99. 10.2307/1229039.

[70] Thomas AJ, Witherspoon KM, Speight SL. “Gendered racism, psychological distress, and coping styles of African American women”, (in eng). Cultur Divers Ethnic Minor Psychol. 2008;14(4):307–14. 10.1037/1099-9809.14.4.307. 18954166

[71] Watson-Singleton NN. Strong Black woman schema and psychological distress: the mediating role of perceived emotional support. J Black Psychol. 2017;43(8):778–88. 10.1177/0095798417732414.

[72] Donovan RA, West LM. Stress and mental health: moderating role of the strong Black woman stereotype. J Black Psychol. 2015;41(4):384–96. 10.1177/0095798414543014.

[73] Williams BR, et al. Marital status and depressive symptoms in African Americans: the moderating role of social and religious resources. J Fam Issues. 2021;42(7):1403–28. 10.1177/0192513x20942819.

[74] I. C. Scarinci, B. M. Beech, W. Naumann, K. W. Kovach, L. Pugh, and B. Fapohunda, “Depression, socioeconomic status, age, and marital status in Black women: a national study,” (in eng), *Ethn Dis,* vol. 12, no. 3, pp. 421–8, Summer 2002. 12148715

[75] Germain CM. Ethnicity, depression, and functional outcomes in older women. Innov Aging. 2019;3(Suppl 1):S605.

[76] Mouzon DM. Can family relationships explain the race paradox in mental health? J Marriage Fam. 2013;75(2):470–85. 10.1111/jomf.12006.

[77] Townsend TG, Kaltman S, Saleem F, Coker-Appiah DS, Green BL. Ethnic disparities in trauma-related mental illness: is ethnic identity a buffer? J Interpers Violence. 2020;35(11–12):2164–88. 10.1177/0886260517701454 29294702

[78] Reed TD, Neville HA. The influence of religiosity and spirituality on psychological well-being among Black women. J Black Psychol. 2014;40(4):384–401. 10.1177/0095798413490956.

[79] E. Jung and M. L. Hecht, “Elaborating the communication theory of identity: identity gaps and communication outcomes,” *Communication Quarterly,* Abstract vol. 52, no. 3, pp. 265–283, Summer2004 2004. [Online]. Available: http://mutex.gmu.edu/login?url=http://search.ebscohost.com/login.aspx?direct=true&db=ufh&AN=15229090&site=ehost-live.

[80] S. Obeng-Gyasi, W. Tarver, R. C. Carlos, and B. L. Andersen, “Allostatic load: a framework to understand breast cancer outcomes in Black women,” (in eng), *NPJ Breast Cancer,* vol. 7, no. 1, p. 100, Jul 30 2021, 10.1038/s41523-021-00309-6. PMC8324921 34330927

[81] Danforth DN Jr. Disparities in breast cancer outcomes between Caucasian and African American women: a model for describing the relationship of biological and nonbiological factors. Breast Cancer Res. 2013;15:1–15. 10.1186/bcr3429 PMC3706895 23826992

[82] Carlos RC, et al. Linking structural racism and discrimination and breast cancer outcomes: a social genomics approach. J Clin Oncol. 2022;40(13):1407–13. 10.1200/jco.21.02004. PMC9851699 35108027

[83] Linnenbringer E, Gehlert S, Geronimus AT. Black-White disparities in breast cancer subtype: the intersection of socially patterned stress and genetic expression. AIMS Public Health. 2017;4(5):526–56. 10.3934/publichealth.2017.5.526. PMC5764177 29333472

[84] Collins KL, Elmore LC. Exploring the deleterious biologic association of stress with cancer disparities in Black women. JAMA Netw Open. 2025;8(2):e2459763–e2459763. 10.1001/jamanetworkopen.2024.59763. 39951272

[85] Black LL, Johnson R, VanHoose L. The relationship between perceived racism/discrimination and health among Black American women: a review of the literature from 2003 to 2013. J Racial Ethn Health Disparities. 2015;2(1):11–20. 10.1007/s40615-014-0043-1. PMC4426269 25973361

[86] Black AR, Woods-Giscombé C. Applying the stress and ‘strength’ hypothesis to Black women’s breast cancer screening delays. Stress Health. Dec2012;28(5):389–96. 10.1002/smi.2464. PMC3696631 23129558

